# Dieting and restrained eating as prospective predictors of weight gain

**DOI:** 10.3389/fpsyg.2013.00577

**Published:** 2013-09-02

**Authors:** Michael R. Lowe, Sapna D. Doshi, Shawn N. Katterman, Emily H. Feig

**Affiliations:** Department of Psychology, Drexel UniversityPhiladelphia, PA, USA

**Keywords:** dieting, restrained eating, weight gain, prevention, obesity

## Abstract

Research in normal weight individuals paradoxically suggests that measures of attempted eating restriction might represent robust predictors of weight gain. This review examined the extent to which measures of dieting (e.g., self-reported weight loss dieting in the past year) and dietary restraint (e.g., the Cognitive Restraint scale from the Three-Factor Eating Questionnaire) have prospectively predicted weight change. We located and reviewed 25 prospective studies containing 40 relevant comparisons. Studies were limited to those in which participants were non-obese (with a mean BMI between 18.5 and 30) and averaged at least 12 years old. Neither measure predicted future weight loss. Fifteen of the 20 comparisons (75%) that examined measures of dieting significantly predicted future weight gain whereas only 1 of 20 (5%) that examined restrained eating measures did so. Two plausible explanations for these findings are that: (1) dieters and restrained eaters do not differ in terms of an underlying proneness toward weight gain, but restrained eating represents a more effective means of preventing it; and (2) normal weight individuals who diet do so because they are resisting a powerful predisposition toward weight gain which dieting ultimately fails to prevent. Recent dieting in non-obese individuals may be a valuable proxy of susceptibility to weight gain. This easily assessed characteristic could identify individuals for whom obesity prevention interventions would be particularly appropriate.

## Introduction

Prevention of weight gain is integral to reversing the obesity epidemic (Childhood Obesity Task Force, 2010). Identifying robust predictors of susceptibility to weight gain would be very helpful but few predictors have been identified. Although it at first appears counter-intuitive, it is possible that efforts to *restrict* food intake might represent valuable indicators of susceptibility to future weight gain. Lowe and Levine (2005) reviewed evidence suggesting that most normal weight restrained eaters restrict their eating to prevent weight gain rather than to lose weight. Stice and colleagues have supported this suggestion by showing that restrained eaters are not in negative energy balance and do not consume less energy in the natural environment than unrestrained eaters (Stice et al., 2004, 2007, 2010). Similarly, Chernyak and Lowe (2010) have shown that the chronic dieting practiced by normal weight restrained eaters (defined by the Restraint Scale (Herman and Polivy, 1980) is motivated by a fear of fatness rather than by a desire to become thin. Because efforts to restrict caloric intake to prevent weight gain are so difficult to consistently maintain, the forgoing evidence suggests that measures of eating restriction in normal weight individuals might represent a proxy of susceptibility to weight gain over the long-term.

There is substantial evidence that dieting and restrained eating are overlapping (Williamson et al., 2007) but nonetheless distinct concepts that relate differently to eating behavior. Multiple laboratory studies (Lowe, 1993, 1995; Giesen et al., 2009; Guerrieri et al., 2009) found sharply diverging reactions to food presentation in dieters compared to restrained eaters. Also, virtually none of the items from frequently-used restraint scales explicitly assess motivations or behaviors aimed at weight loss whereas the term “dieting” (and most of the measures used to assess it) reflects an intention to lose weight (Lowe and Thomas, 2009). Therefore, one possible and potentially critical distinction between these constructs appears to be the *purpose* of restricting caloric intake, *viz.* to avoid gaining, as opposed to losing, weight. We therefore examined separately studies that measure dieting and restrained eating as prospective predictors of weight change.

We located 25 studies involving 40 comparisons that assessed measures of dieting, restrained eating or both as predictors of subsequent weight change among groups whose mean BMI was less than 30 kg/m^2^. We restricted our review to such studies because prevention of weight gain in those who have not become obese is a particularly compelling objective, as many of the health problems associated with higher weight are most likely to occur when BMI reaches 30 or higher (Flegal et al., 2013).

The purpose of our analysis was to determine whether one or both types of measures were related to subsequent weight change and, if so, to assess how frequently each type predicted weight change. We examined the simple percentage of studies in each category that significantly predicted future weight change. Our goal was to review studies of restrained eating and dieting in samples where the average BMI of participants was under 30 to determine the extent to which these measures predicted future weight change, as well as the direction of the weight changes predicted.

## Methods

### Study retrieval and selection

Studies were identified by conducting searches of the PsycINFO, Medline and PubMed databases. Two types of keywords were combined to conduct searches in each database. The first group included terms related to weight change over time (i.e., *prospective, weight gain, weight change*). The second group included terms related to the measures of interest (i.e., *restraint, dieting, cognitive restraint, dietary restraint, restrained eating, Restraint Scale, Three Factor Eating Questionnaire, Dutch Eating Behavior Questionnaire*, and *Dutch Restrained Eating Scale*). Reference lists of retrieved articles were also reviewed to determine if any additional articles would add to our selection of articles to review.

### Inclusion and exclusion criteria

Only truly prospective studies (i.e., studies that examined some measure of dieting or restraint at Time 1 and weight at Time 1 and 2) were examined to limit our review to studies that can determine temporal precedence of restraint or dieting in the predictive relationships. We included studies that used one of three types of restraint measures: the Revised Restraint Scale (Polivy et al., 1988) and the restraint subscales from the Three Factor Eating Questionnaire [aka Eating Inventory (Stunkard and Messick, 1985)] and the Dutch Eating Behavior Questionnaire (Van Strien et al., 1986). In the articles that examined dieting, dieting was measured in three different ways noted in Table 2. In some articles, participants were asked if they were currently on a diet to lose weight (i.e., current dieting). In others, they were asked about frequency of dieting within the past year (i.e., recent dieting). Studies examining weight cycling (number of times a certain amount of weight has been lost over a lifetime) were excluded to limit our paper to current or recent attempts at dietary restriction. One study (Pietiläinen et al., 2012) was included even though it only examined lifetime history of dieting because participants were only 16 years old at the start of the study. The chances that they had begun dieting long before the prior year are low. Two studies examined history of dieting and current dieting (French et al., 1994; Lowe et al., 2006). Because most of the other dieting studies measured current dieting the results for current dieting from these two studies were used here. Finally, only studies that examined weight change as a continuous outcome were included.

To narrow the focus of our paper to adolescents and adults, studies of children with an average age under 12 years were not included. Additionally, studies were only included in which participants had a mean body mass index (BMI) that fell in the non-obese weight range (i.e., BMIs between 18.5 and 30).

### Statistical analysis

Our primary analysis involved a calculation of the proportion of analyses that predicted weight change across the different methods of measurement. Although we considered using the Liptak-Stouffer Z-method (which adjusts for each study's sample size when examining the strength of its predictive effect) to compare the ability of restraint and dieting measures to predict future weight change, a large proportion of analyses were missing information regarding exact *p*-values that are needed to calculate this statistic. Additionally, a meta-analysis could not be conducted because measures of effect size were not available for the majority of analyses. In the cases where analyses were done separately for males and females or by age groups, each analysis within a study was counted separately since these represent independent tests of prediction (Klesges et al., 1992; French et al., 1994; Korkeila et al., 1999; Juhaeri et al., 2001; Drapeau et al., 2003; Field et al., 2003; Tiggemann, 2004; de Lauzon-Guillain et al., 2006; Neumark-Sztainer et al., 2006; Field et al., 2007).

## Results

The shortest time period in which weight change was assessed was five and a half months, and the longest time period was 9 years. However, the amount of time that transpired between the two measurements of weight was usually greater than 1 year. This is desirable because a relatively long period of time is necessary to see a systematic change in weight. There were enough studies in both tables that were 1 year or longer and had substantial sample sizes so we can be confident that if meaningful changes in weight were occurring, they likely would have been detected statistically.

Table 1 includes studies of dietary restraint as predictors of weight change and Table 2 includes studies of dieting as a predictor of weight change (See Tables 1, 2 for references).

**Table 1:** **Prospective studies using restraint measures to predict weight change**.

**Citation**	***N***	**Sex**	**Age**	**TI BMI**	**Measure type**	**BMI: SR or PM**	**Time length**	**Predict weight gain? Y or N**
de Lauzon-Guillain et al., 2006	Younger: 271	*M* = 139	Younger: 16.4^a^	Younger: 20.1	CR^b^	PM	T1—T2: 2 yrs	N—Younger
	Older: 466	*F* = 132	Range: 14–24	Older: 24.7				N—Older
		*M* = 207	Older: 42.7					
		*F* = 259	Range: 31–67					
Delinsky and Wilson, 2008	149	F	17.92 (±0.50)	22.3 (±3.52)	DR	SR	T1—T2: 8 mos	N
Drapeau et al., 2003	75	*M* = 30	M: 44.2 (±2.6)	M: 29.7 (±1.3)	CR	PM	T1—T2: 6 yrs	N—M
		*F* = 45	F: 38.0 (±2.0)	F: 28.3 (±1.1)				N—F
Finlayson et al., 2012	120	*M* = 22	19.2 (±2.6)	21.9 (±3.2)	CR^b^	PM	T1—T2: 12 mos	N
		*F* = 98						
Hays et al., 2006	36	F	61.3 (±3.1)	23.5 (±3.1)	CR	PM	T1—T2: 4.4	N
			Range: 55–65				(±0.9) yrs	
Klesges et al., 1992	250	*M* = 123	35.7 (±4.51)	M: ~26.95	RS	PM	T1—T2: 1 yr	N—M
		*F* = 127	Range: 26–53	F: ~24.73				Y—F
Klesges et al., 1991	305	*M* = 98	21 (±6.23)	Overall means not reported	RS	SR	T1—T2: 2.5 yrs	N
		*F* = 207						
Koenders and van Strien, 2011	1562	*M* = 963	44.10 (±8.90)	25.08 (±3.5)	DR	SR	T1—T2: 2 yrs	N
		*F* = 599						
Lowe et al., 2006	69	F	18.06 (±0.23)	21.9 (± 2.4)	CR & DR	PM	T1—T2: 8 mos	N
			Range: 18–19					
Pliner and Saunders, 2008	72	*M* = 15	~18.7	M: ~23.5	RS	PM	T1—T2: 5.5 mos	N
		*F* = 57		F: ~22.9				
Savage et al., 2009	163	F	35.7 (±4.7)	26.5 (±6.2)	CR	PM	T1—T2: 6 yrs	N
Snoek et al., 2008	808	*M* = 405	Younger^c^: 13.4 (±0.50)	Younger: ~18.9	DR	SR	T1—T2: 3 yrs	N^d^
		*F* = 403	Older: 15.2 (±0.60)	Older: ~19.9				
			Range: 13–16					
Stice et al., 1999	692	F	14.9	21.9	RS	PM	T1—T2: 4 yrs	N
			Range: 3.6–17.1					
Tiggemann, 2004	77	*M* = 19	25.12 (±8.76)	Not reported	RS	SR	T1—T2: 8 yrs	N—M
		*F* = 58						N—F
Tucker and Bates, 2009	192	F	40 (±3)	Not reported	DR	PM	T1—T2: 3 yrs	N
Van Strien et al., 1986	590	*M* = 308	48.2 (±14.5)^e^	~26.74 (±4.33)	DR	SR	T1—T2: 2 yrs	N
		*F* = 282	Range: 19–75					

T1, Time 1 measurement; T2, Time 2 measurement; BMI, body mass index; DR, Dutch Eating Behavior Questionnaire, Restrained Eating subscale; CR, Three Factor Eating Questionnaire, Cognitive Restraint subscale; RS, Herman and Polivy Revised Restraint Scale; SR, self-report; PM, physical measurement.

a This study included adult parents and their offspring. Each group was analyzed separately.

b These studies used the cognitive restraint scale from the 18-item revised Three-Factor Eating Questionnaire (38), whereas all other studies labeled CR used the cognitive restraint scale from the original TFEQ (15).

c Participants were sibling pairs; data were split by cohort (older sibling and younger sibling) and analyzed separately; non-independent observations are not an issue because older siblings were compared only to other older siblings in other families. Sibling pairs were not combined in analyses.

d This study looked at 4 longitudinal analyses across 3 years (year 1 to year 2; year 2 to year 3). Since only one of these analyses predicted weight gain from year 1 to year 2, we counted this study as having not found that restraint predicted weight gain over the 3 years.

e Age information applies to all participants included in baseline assessments, not just those who completed follow up.

**Table 2:** **Prospective studies using dieting measures to predict weight change**.

**Citation**	***N***	**Sex**	**Age**	**TI BMI**	**Measure type**	**BMI: SR or PM**	**Time length**	**Predict weight gain? Y or N**
Delinsky and Wilson, 2008	149	F	17.92 (±0.50)	22.3 (±3.52)	Current	SR	T1—T2: 8 mos	N
Field et al., 2003^a^	14972	*M* = 6769	M: 11.9 (± 1.5)	M: 19.1 (±3.3)	Recent	SR	T1—T2: 1 yr	Y—F
		*F* = 8203	F: 12.0 (±1.6)	F: 19 (±3.3)			T2—T3: 1 yr	Y—M
			Range: 9–14				T3—T4: 1 yr	
Field et al., 2007	8402	*M* = 4100	M: 15 (±0.1)	M: 22.2 (±0.1)	Current^b^	PM^c^	T1—T2: ~1 yr	Y—F
		*F* = 4302	F: 14.9 (±0.1)	F: 22.0 (±0.1)			T2—T3: ~5 yrs	N—M
			Range: 11–20					
French et al., 1994	3671	*M* = 1639	M: 39.1 (±9.8)	25.8 (±4.9)	Current^d^	PM	T1—T2: 2 yrs	Y—F
		*F* = 1913	F: 37.3 (±10.7)					N—M
Juhaeri et al., 2001	10554	*M* = 4689	Overall means not provided	Overall means not provided	Current	PM	T1—T2: 3 yrs	Y - F
		*F* = 5855					T2—T3: 3 yrs	Y - M
Korkeila et al., 1999^e^	7729	*M* = 3536	Younger: 18–29	Overall means not provided	Current	SR	T1—T2: 6 yrs	Y—Younger F
		*F* = 4193	Older: 30–54					Y—Younger M
								Y—Older F
								Y—Older M
Lowe et al., 2006	69	F	18.06 (±0.23)	21.9 (±2.4)	Current	PM	T1—T2: 8 mos	Y
Neumark-Sztainer et al., 2006^f^,^g^	2516	*M* = 1130	C1^h^: 12.8 (±0.8)	T1 BMI not reported	Recent	T1: PM,	T1—T2: 5 yrs	Y—F
		*F* = 1386	C2: 15.8 (±0.8)	T2: *F* = 23.9 (±5.1)		SR		Y—M
				*M* = 24.6 (±4.8)		T2: SR		
Pietiläinen et al., 2012^i^	4129	*M* = 1933	16	M: 20.4 (20.3–20.6)	Lifetime^j^	SR	T1—T2: 9 yrs	Y
		*F* = 2207		F: 20.2 (20.1–20.4)				
Savage et al., 2009	163	F	35.7 (±4.7)	26.5 (±6.2)	Current	PM	T1—T2: 6 yrs	Y
Senf et al., 2006	1081	F	Grade 6–9	0.62 (±1.02) BMI	Recent	PM	T1—T2: 1 yr	N
				z-scores			T2—T3: 1 yr	
							T3—T4: 1 yr	
Stice et al., 2004, 2007, 2011, 2010	692	F	14.9	21.9 (±4.0)	Current	PM	T1—T2: 4 yrs	N
			Range: 13.6–17.1					

T1, Time 1 measurement; T2, Time 2 measurement; T3, Time 3 measurement; T4, Time 4 measurement; BMI, body mass index; Current, current dieting was assessed (e.g., Are you on a diet right now?); Recent, How much have you dieted in the past year?; Lifetime, lifetime dieting was assessed (e.g., How many times have you intentionally lost weight in your lifetime?) SR, self-report; PM, Physical measurement.

a Same data as in Field et al., 2004 (39) so only one study was included.

b This definition included people who said they were currently dieting at Time 1 or 2.

c Height and weight were self-reported at Waves I, II, and III, and were also measured at Waves II and III. Correlation between self-reported and measured weights (r = 0.96) and BMI (r = 0.94) was high. Measured weights were used whenever they were available.

d Results were also given for past dieting (i.e., measured as answering yes to “previously dieted to lose weight” or “previous participation in a formal weight loss program”) but for the purposes of the current study, only current dieting results were used.

e Analyses were done separately by sex and age group.

f Same data as in Neumark-Sztainer et al., 2007 (40) so only one study was included.

g Neumark-Sztainer et al., 2012 found consistent results when extending follow-up out 10 years.

h C1 and C2 are labels given to different cohorts in the study.

i All participants were pairs of twins.

j Lifetime dieting was assessed, however since all participants were only 16 at baseline, this is likely equivalent to a question of recent dieting in adults.

With the exception of one study of post-menopausal women (Hays et al., 2006) where no significant change in weight occurred, all studies reported an average weight gain. Thus, consistent with secular trends, the majority of the prospective studies found that body weight on average went up over time.

For the analyses that included measures of restrained eating as predictors of weight change, one of the 20 analyses (5%) found restraint measures to predict weight gain, whereas 19 analyses found no significant predictive relationships (See Table 1 for details). For the analyses that included measures of dieting as predictors, 15 analyses (75%) found that dieting status predicted weight gain and five analyses did not (See Table 2 for details).

On average the dieting-related studies had larger sample sizes than the restraint studies, creating a potential source of bias. However, it is also noteworthy that five studies using restraint measures that had sample sizes ranging from medium to very large (141, 149, 271, 692, 808, and 1562) found no prediction of weight gain.

## Discussion

Identifying predictors of weight gain is an area of great importance for public health. Results of 20 analyses involving restrained eating and 20 others involving dieting revealed two main findings. First, neither measures of restrained eating nor dieting ever predicted better weight control (i.e., weight loss or less weight gain). Second, when degree of weight gain was successfully predicted, dieting predicted it much more consistently than measures of restraint. This finding was supported by proportion analyses (dieting predicted weight gain in 75% of these analyses while restrained eating measures predicted weight gain in 5% of analyses).

It is important to note that studies that measured dieting generally had larger sample sizes than the studies of restrained eating, so the studies of dieting had more power to detect predictive effects dieting if they existed. Conceivably, if the sample sizes in the restraint studies were comparable to those in the dieting studies, no difference in prediction would have emerged. Nonetheless, as the data stands, restraint measures did not predict weight gain even with sample sizes of greater than 1500 individuals (Koenders and van Strien, 2011), and dieting measures did predict gain even with samples as small as 69 participants (Lowe et al., 2006). It is also worth noting that measures of restraint are multi-item scales while dieting measures are single item measures. Single item measures typically have less power to detect effects because there is less variability in their scores; however, the predictive ability of single item dieting measures was actually greater than restraint in the current analysis. Taken together, findings suggest that in non-obese individuals, measures of attempts at dietary restriction predict weight gain rather than weight loss and measures of dieting are more robust predictors of this gain than measures of restrained eating.

We have written at length about the differences between restrained eating and dieting (Lowe and Levine, 2005) but considerable confusion continues to surround these terms. One hypothesis is that restrained eaters are perhaps best characterized as “weight watchers” who are concerned about their food intake and try to limit intake, particularly of energy dense foods. Most restrained eaters are not currently on a diet (Lowe, 1993) and as a group they do not restrict their intake enough to lose weight (Stice et al., 2004, 2007, 2010). Dieters, on the other hand, are assumed to have a goal of restricting their caloric intake sufficiently to lose weight. In laboratory settings, when dietary control is challenged by the introduction of disinhibiting conditions, current dieters restrict their food intake much more successfully than restrained eaters who are not currently dieting (Lowe, 1993, 1995; Giesen et al., 2009; Guerrieri et al., 2009). The results of the present study, however, suggest that this short-term control may mask an underlying susceptibility toward over-consuming energy and gaining weight.

The prospective nature of these studies confirms that dieting at one point in time is likely to predict weight gain at a later point in time, but their correlational nature prevents causal conclusions from being drawn.

Lowe and Levine (2005) provide one possible explanation as to why dieters are prone to future weight gain. Individuals who are gaining weight are more likely to go on weight loss diets than those who are not gaining weight, but evidence indicates that weight lost on a diet is usually regained (Wadden et al., 2004). Therefore, individuals who are prone toward weight gain are not only likely to go on weight loss diets but are likely to do so repeatedly. Although weight loss reduces metabolic rate and may contribute to the regain of previously lost weight, there is little reason to believe that a *history* of weight loss dieting contributes to weight gain beyond the weight dieters eventually regain when returning toward their pre-dieting weight. From this perspective, having a history of weight loss dieting comprises a proxy of susceptibility toward weight gain from multiple (genetic, environmental, etc.) causes but not from past dieting itself. It is of course possible that past bouts of weight loss dieting do help cause the likelihood of future weight gain, but available evidence suggests that among non-obese individuals (like those studied here), weight loss and regain may cause body composition to shift toward an increased body fat percentage, but not to an increase in body mass *per se* (Dulloo et al., 2012). The fact that restrained eating is not related to future weight could be due to restrained eaters having less of a predisposition toward weight gain or to their engagement in more frequent or more effective weight gain prevention behaviors.

The term “dieting” also requires further specification because this term could apply to a wide range of practices, from eating more fruits and vegetables to lose weight (a generally healthy practice) to repeatedly fasting for lengthy periods [a practice that has been linked to increased risk of developing eating disordered behaviors (Stice et al., 2008)]. One recent study (Savage and Birch, 2010) found differences between individuals with healthy and unhealthy dieting practices. Specifically, dieters who engaged in unhealthy weight loss strategies gained significantly more weight than non-dieters or dieters who used healthy weight loss strategies. However, another study found that frequency of past dieting predicted future weight gain whereas frequency of engaging in unhealthy dieting behaviors did not (Field et al., 2010). These results give conflicting evidence on whether it is going on weight loss diets *per se*, or the particular behaviors that dieters may practice that represent the risk factor for future weight gain. Given these discrepant findings, the role of dieting as either a proxy for or cause of future weight gain requires further research.

Limitations to the current study do exist. Some of the studies used self-report to assess body weight and height. Self-report measures are less desirable because they are less accurate, but fortunately more than half of the studies included physical measures of height and weight, and there were no obvious differences in terms of which studies used self-report vs. measurement. These facts bolster confidence that the pattern of findings observed accurately reflect actual changes in body weight. Another limitation regards generalizability of findings. In regard to gender, although there were more females than males in the studies examined, both genders are fairly well-represented such that conclusions drawn can be reasonably applied to individuals regardless of gender. In addition, although it is not listed in the tables, minorities were not well-represented among studies. The majority of the studies included mostly Caucasian individuals. Therefore, these findings may not be generalizable to different ethnic minority groups. Lastly, the current study only examined individuals in the normal and overweight weight ranges and therefore findings should be generalized to other populations with caution.

The potential risk of bias due to sample size discrepancies is third limitation. However, as mentioned above, even the restraint study with the highest sample sizes found that restraint was not predictive of weight gain, whereas a study with one of the smallest sample sizes found that measures of current and past dieting did predict weight gain while a measure of restraint did not. Larger scale studies are needed to evaluate whether or not measures of restraint are capable of predicting of weight gain.

Future research should include more studies of restraint measures, done with larger sample sizes, in order to confirm that restraint measures are weak or inconsistent predictors of weight gain. Because the Restraint Scale assesses past dieting more explicitly than the TFEQ and DEBQ restraint measures, future research should also test the hypothesis that the Restraint Scale would be more predictive of future weight gain than the other two scales. Future studies should also include effect sizes so that meta-analyses can be conducted. Finally, because most of the studies we reviewed were comprised mostly of adolescents and younger adults, additional studies of older adults are needed.

Current or past dieting behavior, which is easily assessed, could provide a “shortcut” for identifying individuals for whom future weight gain is likely. Assessing dieting in the recent past (e.g., in the past year) might predict subsequent weight gain better than measures that assess dieting over one's lifetime (for example, measures of weight cycling that assess the historical frequency of significant weight losses). That is, if dieting is a proxy of susceptibility to weight gain, then the more recent the practice of dieting the more likely it will reflect a current struggle against weight gain. Such measures have the potential to be effective indicators of normal weight individuals who are likely to gain weight over time. Future research should examine this possibility further and should also examine other behavioral or biological indicators of weight gain that might predict weight gain even more robustly. The development of accurate measures that predict future weight gain is essential for the identification of individuals who might benefit most from programs to counteract an underlying predisposition toward weight gain.

There is initial evidence that various ecological and parental interventions show statistically significant effects on weight gain prevention in children (Doak et al., 2006; Nixon et al., 2012). However, clinically meaningful weight gain prevention programs will eventually need to demonstrate long-lasting preventive effects. Furthermore, research has shown that weight suppression—i.e., the difference between one's previous highest weight at adult height and current weight—is a robust predictor of weight gain over short- and long-term follow-ups (e.g., Lowe et al., 2006; Herzog et al., 2010; Stice et al., 2011). This research suggests that once an elevated body weight has been reached, it may be very difficult to permanently return to a lower weight. Indeed, findings imply that individuals who reach weights significantly above their current weights may be caught in a biobehavioral bind in that successful weight loss dieting (which increases weight suppression) may forestall but not prevent a return toward their past highest weights.

### Conflict of interest statement

The authors declare that the research was conducted in the absence of any commercial or financial relationships that could be construed as a potential conflict of interest.
